# INTEGRATING PRACTITIONER KNOWLEDGE INTO AN ALGORITHM TO IDENTIFY CASES FOR REVIEW BY AN ADULT MALTREATMENT MDT

**DOI:** 10.1093/geroni/igad104.0608

**Published:** 2023-12-21

**Authors:** Erin Thayer, Julia Martinez, Lori Mars, Zach Gassoumis

**Affiliations:** University of Southern California - Keck School of Medicine, Los Angeles, California, United States; University of Southern California Keck School of Medicine, Los Angeles, California, United States; USC, Alhambra, California, United States; University of Southern California, Los Angeles, California, United States

## Abstract

Case review multidisciplinary teams (MDTs) bring together professionals from health, social service, and justice systems to address complex cases of elder abuse, neglect, self-neglect, and maltreatment of younger adults with disability or vulnerability. MDTs are a promising intervention, but MDTs nationwide face challenges identifying cases for review. In partnership with San Francisco Adult Protective Services (APS), we developed an algorithm-driven process to systematically identify APS cases that appear appropriate for MDT review. To construct the algorithm, a retrospective analysis of APS case data was augmented by insights received during six listening sessions held with 48 study partners (MDT members and APS staff). These sessions gauged perspectives on cases’ suitability for MDT review. Listening session transcripts were coded using constant comparative analysis, revealing themes of Client, Case Details, and Service Utilization Patterns. Client included physical or cognitive impairment, high-risk of abuse or other harms, need for assistance from multiple service systems, and limited motivation to make changes. Case Details referred to aspects of the abuse such as concurrent self-neglect, client protectiveness of the suspected abuser, client denial of the abuse, and limited evidence of criminal conduct. Service Utilization Patterns were recurrence within APS, attempting multiple solutions, inability to provide services, and referral by medical professionals, fire departments, or paramedics. These topics guided the inclusion of variables in the project’s algorithms. This mixed-methods approach provides an example of community-engaged research being applied to develop knowledge and guide the practice of service delivery for vulnerable older adults and other victims of adult maltreatment.

